# Ternary SnS_2–*x*_Se_*x*_ Alloys Nanosheets and Nanosheet Assemblies with Tunable Chemical Compositions and Band Gaps for Photodetector Applications

**DOI:** 10.1038/srep17109

**Published:** 2015-11-30

**Authors:** Jing Yu, Cheng-Yan Xu, Yang Li, Fei Zhou, Xiao-Shuang Chen, Ping-An Hu, Liang Zhen

**Affiliations:** 1 School of Materials Science and Engineering, Harbin Institute of Technology, Harbin 150001, China; 2 MOE Key Laboratory of Micro-systems and Micro-structures Manufacturing, Harbin Institute of Technology, Harbin 150080, China; 3 Department of Physics, Harbin Institute of Technology, Harbin 150001, China

## Abstract

Ternary metal dichalcogenides alloys exhibit compositionally tunable optical properties and electronic structure, and therefore, band gap engineering by controllable doping would provide a powerful approach to promote their physical and chemical properties. Herein we obtained ternary SnS_2−*x*_Se_*x*_ alloys with tunable chemical compositions and optical properties via a simple one-step solvothermal process. Raman scattering and UV-vis-NIR absorption spectra reveal the composition-related optical features, and the band gaps can be discretely modulated from 2.23 to 1.29 eV with the increase of Se content. The variation tendency of band gap was also confirmed by first-principles calculations. The change of composition results in the difference of crystal structure as well as morphology for SnS_2−*x*_Se_*x*_ solid solution, namely, nanosheets assemblies or nanosheet. The photoelectrochemical measurements indicate that the performance of ternary SnS_2−*x*_Se_*x*_ alloys depends on their band structures and morphology characteristics. Furthermore, SnS_2−*x*_Se_*x*_ photodetectors present high photoresponsivity with a maximum of 35 mA W^−1^ and good light stability in a wide range of spectral response from ultraviolet to visible light, which renders them promising candidates for a variety of optoelectronic applications.

Two-dimensional (2D) layered metal dichalcogenides nanomaterials are attracting intense interest due to their fascinating properties and potential applications in optics, optoelectrinics, catalysis, energy conversion and storage, etc^1^,^2^,^3^,^4^,^5^,^6^,^7^,^8^. The dichalcogenides possess individual sandwiched X−M−X layer structure with weak out-of-plane van der Waals forces between molecular layers and strong in-plane chemical bonding within the layers. Unlike graphene with zero band gap, metal dichalcogenides 2D layered structures own sizable band gaps and exhibit strong light-matter interaction, which are promising for electronic and optoelectronic devices^9^. It is required to modify pristine materials to promote their physical and chemical properties. Chemical doping is a high-efficiency approach to fine-tune the structures and optical features of these layered materials. In view of the similar atomic structure of congeners, it is possible to construct a mixed alloy system (MSSe, M is metal atom) with tunable composition and continuously tuned band gap, which has been widely studied for applications in nanoelectronics and nanophotonics^10^,^11^,^12^,^13^. For example, optical band gap modulations have been reported *via* a broad range of Se doping of atomic thin MoS_2_ on SiO_2_ by chemical vapor deposition (CVD) method. The band gaps of ternary alloys could be finely tuned between 1.85 and 1.60 eV with the change of Se concentration^14^. The photoelectric properties of monolayer MoS_2(1−*x*)_Se_2*x*_ devices have been studied, which largely depends on the chemical composition of ternary alloys. Compared with S-rich ones with decreased diffusion approach of photogenerated carriers, the photocurrents present significant decrease for Se-rich alloys^15^. Theoretical calculations have proved that mixing energy of transition metal dichalcogenides (TMDs) ternary alloys is low and mixed MoS_2_/MoSe_2_/MoTe_2_ compounds are thermodynamically stable at room temperature. Moreover, their compositions and band gaps could be continuously tuned between the constituent limits, indicating the benefit of band gap engineering for optoelectronic applications^16^.

As narrow-gap IV–VI semiconductors, tin dichalcogenides have been widely studied for electronic and optoelectronic applications^17^,^18^,^19^,^20^. Their valence bands (VB) primarily depend on the *p* orbital of chalcogens, while the conduction bands (CB) are hybridized orbitals generated by the interaction of *p* orbital of chalcogens and *s* orbital of tin atom^21^. Layered SnS_2_ and SnSe_2_ are both isostructural with typical CdI_2_−type structure and possess indirect band gaps of 2.18−2.44 eV and 1−2 eV, respectively^19^,^22^. Consequently, it is feasible to form a solid solution of isostructural SnS_2−*x*_Se_*x*_ alloys. This might be an efficient approach to the strong incorporation and homogeneous distribution of different atoms, which are favorable for the separation of photo-generated charges. The band gap engineering would offer an efficient platform for changing the electronic performance of tin dichalcogenides. For example, SnS_2−*x*_Se_*x*_ single crystals have been obtained *via* chemical vapor transport with iodine as transport agent. Increasing Se content could lead to increase of dielectric constant and the decrease of donor ionization energy, and suppress the gate-modulated drain-source current in field effect transistors (FETs)^21^. Hadjiev *et al.* reported the variations of Raman phonon frequency and line-width with the change of Se content in mixed crystals SnS_*x*_Se_2−*x*_ *via* experimental method and density functional perturbation theory (DFPT) first-principle calculations. The absence of overlapping of the corresponding phonon dispersion bands in SnS_2_ and SnSe_2_ results in the two-mode behavior (2MB) of A_1g_ and E_g_ vibrations of Se (S) atoms^23^. The modified materials based on 2D SnS_2_ crystals would provide diversified strategies for electronic structure engineering and efficient device applications in electronics and optoelectronics.

Previous works about 2D MSSe are mainly focusing on CVD growth^24^,^25^,^26^, however, there have been only a few works on controlled synthesis of MS_*x*_Se_*y*_ alloys based on solution approach, which provides a efficient method for large-scale preparation and promising application. In addition, the photoelectronic properties of SnS_2−*x*_Se_*x*_ alloys with different chemical compositions have been rarely reported according to our knowledge. In this work, composition-tunable SnS_2−*x*_Se_*x*_ alloys were successfully prepared by a one-step solvothermal procedure. Upon Se doping, the variations of crystal structures and morphologies of SnS_2_ nanosheets were investigated. Raman scattering, UV-vis-NIR absorption spectra and first-principles calculations were carried out to reveal their composition-dependent optical properties. The photoelectrochemical performances of SnS_2−*x*_Se_*x*_ alloys under the irradiation of green light (*λ* = 550 nm) were examined. We fabricated SnS_2−*x*_Se_*x*_ devices to study their optoelectronic properties as photodetectors. The electrical properties of different devices were characterized. The photoresponsivity of SnS_0.44_Se_1.56_ films was found to be strongly dependent on incident light power and wavelength.

## Results

We first examined the chemical compositions of SnS_2−*x*_Se_*x*_ alloys according to EDS analysis, as shown in Supplementary Fig. S1 and Table 1. The actual concentrations of S and Se atoms in SnS_2−*x*_Se_*x*_ alloys were close to the nominal concentration. Moreover, the ratios of (S + Se)/Sn in SnS_2−*x*_Se_*x*_ samples were close to the stoichiometry of 2. EDS elemental mapping of SnS_0.82_Se_1.18_ alloy (in Supplementary Fig. S2) clearly reveals homogenous composition distribution of Sn, S and Se elements.

**Figure 1:**
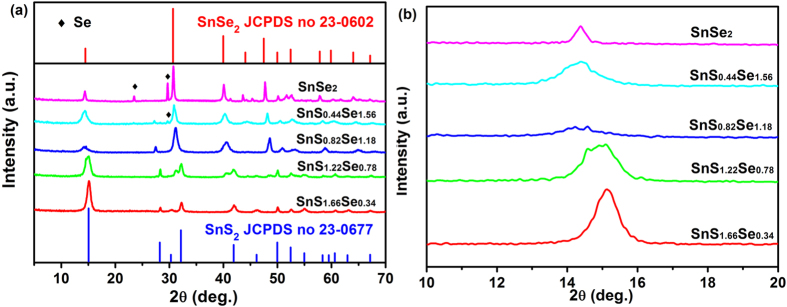
(**a**) XRD patterns of SnS_2−*x*_Se_*x*_ alloys with different Se contents. (**b**) Enlarged patterns of (**a**) from 10 to 20 degrees of SnS_2−*x*_Se_*x*_ alloys.

XRD analysis for SnS_2−*x*_Se_*x*_ alloys with different Se contents was performed to examine the change of crystal structure upon Se doping. As shown in Fig. 1, SnSe_2_ can be indexed with hexagonal CdI_2_-type unit cells (JCPDS no. 23-0602). The lattice constants of hexagonal SnS_2_ are *a* = *b* = 3.649 Å and *c* = 5.899 Å (JCPDS no. 23-0677), and the values of SnSe_2_ are *a* = *b* = 3.81 Å and *c* = 6.14 Å. As expected, the main peak positions of SnS_2−*x*_Se_*x*_ alloys gradually shift toward lower angles with increasing Se content (Fig. 1b), indicating the increase of lattice constants and formation of solid solution rather than the mechanical mixture of two pure phases^27^,^28^. The continuous peak shifting (lattice expanding) of ternary alloys might rule out the phase separation or separated nucleation of SnS_2_ or SnSe_2_ nanomaterials^29^,^30^. As shown in Supplementary Fig. S3 and Table 1, the change of lattice parameter *a* in SnS_2−*x*_Se_*x*_ alloys is in linear with the change of Se content. According to Végard’s Law, the variation of lattice parameters of ternary alloys would present a linear relationship with composition in the absence of strong electronic effects^28^. Consequently, the variation tendency in SnS_2−*x*_Se_*x*_ is in agreement with the Végard’s Law and demonstrates the formation of homogeneous alloy structure^31^. In additional, the crystallite dimensions of all the samples were calculated by Scherrer equation, which were 12.4 nm, 9.9 nm, 11.5 nm, 12.0 nm, 7.9 nm, and 23.6 nm with the increase of Se contents, respectively (Supplementary Table S1).

**Figure 2:**
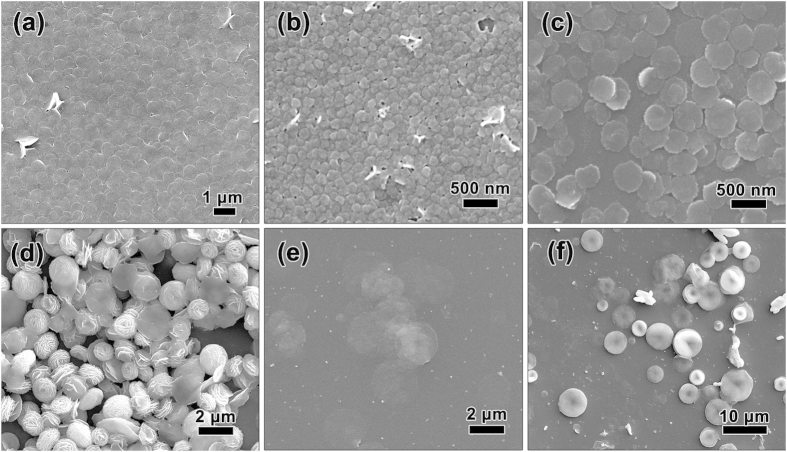
Typical SEM images of SnS_2−*x*_Se_*x*_ alloys with different Se concentrations. (**a**) SnS_2_; (**b**) SnS_1.66_Se_0.34_; (**c**) SnS_1.22_Se_0.78_; (**d**) SnS_0.82_Se_1.18_; (**e**) SnS_0.44_Se_1.56_; (**f**) SnSe_2_.

The morphology variation of SnS_2−*x*_Se_*x*_ alloys with Se contents was shown in Fig. 2, and the corresponding AFM and height curves were provided in Supplementary Fig. S4. Similar to our previous work^32^, pure SnS_2_ presented typical nanosheets structure with lateral sizes of ca. 0.8−1 μm and thicknesses of ca. 22 nm. The introduction of Se element would have a large affect on the morphology of the samples. Upon Se doping, nanosheets and nanosheet assemblies are formed, the later one of which consists of building block of nanosheets. When low content of Se element was introduced (*x* = 0.34), small NSs structure were obtained with lateral dimensions of ca. 80−160 nm and thicknesses of ca. 10−20 nm. With the increase of Se concentration, SnS_1.22_Se_0.78_ showed nanosheet shape with lateral sizes of around 400−600 nm and thicknesses of around 20−30 nm. When the value of *x* was 1.18, the sample would form into stacked structure (1−2 μm) composed of numerous 2D nanosheets. Interestingly, SnS_0.44_Se_1.56_ alloy owned ultrathin nanosheets structure with diameters of ca. 1.8−2.5 μm and thicknesses of ca. 8 nm with further increase of Se content (*x* = 1.56). The pure SnSe_2_ sample showed 2D layered plates structure with large sizes of several micrometers and heights of hundreds of nanometers. As shown in Fig. 2f and Supplementary Fig. S4g, SnSe_2_ plates were assembled by tens of individual nanosheets and the thickness of nanosheet was determined to be around 25.4 nm. The thickness variation tendency of SnS_2−*x*_Se_x_ nanosheets was approximately consistent with the crystallite sizes derived from Scherrer equation. The TEM images and SAED patterns were provided in Supplementary Fig. S5. The nanosheets structure of SnS_1.22_Se_0.78_, SnS_0.44_Se_1.56_, and SnSe_2_ were in good agreement with the SEM and AFM results. Remarkably, the pure SnS_2_^32^ and SnSe_2_ are single crystalline and own 2D layered structure with hexagonal symmetry. However, the diffraction rings of polycrystalline would appear with the introduction of Se element. The tunable composition may provide a good candidate for photodector applications. As we know, the crystal growth habits and environmental factors would play a critical role in crystallization process^33^. Layered SnS_2_ and SnSe_2_ are both isostructural with typical CdI_2_−type structure. According to our previous work^32^, the synthesis approach in this work would provide a favorable environmental to induce tin dichalcogenides to grow along lateral direction and expose (001) facets. Consequently, we believe the ternary SnS_2−*x*_Se_*x*_ alloys would prefer to grow and form 2D nanosheets structure. However, the practical growth environment may affect the self-assembling behavior. The (001) orientation is preferentially oriented for pure SnS_2_^32^, and that of SnSe_2_ is (101) facet^19^. The different crystal orientation might result in synergistic effect on the crystal growth of ternary alloys. The competition phenomenon was especially obvious in SnS_0.82_Se_1.18_ nanosheets assemblies, which owned nearly equal S and Se concentrations in the initial stage of chemical reaction (in view of the incomplete dissolution of add Se). Before the solvothermal reaction, all of the reactants were dissolved in the TEG. However, it is difficult to understand the exact reaction mechanism during such a fast reaction. The detailed mechanism of nanosheets and nanosheets assemblies is still under investigation.

**Figure 3:**
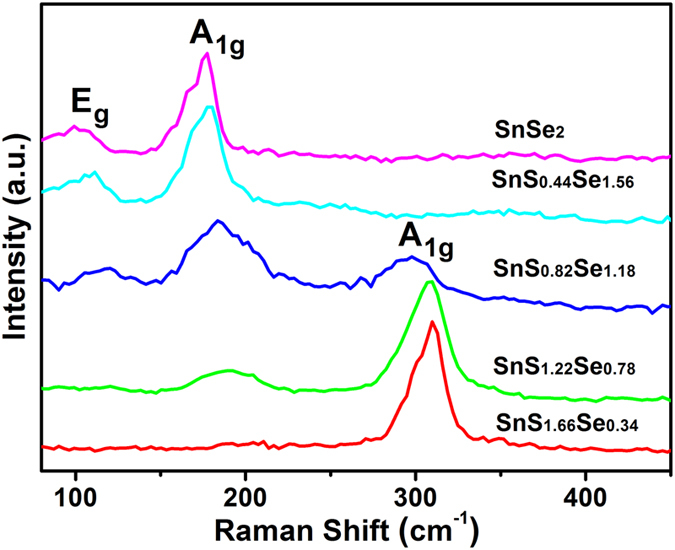
Raman spectra of SnS_2−*x*_Se_*x*_ alloys with different *x* values.

Raman spectra were used to examine the composition-dependent vibration modes of SnS_2−*x*_Se_*x*_ ternary alloys. Figure 3 presents the normalized Raman spectra of as-prepared SnS_2−*x*_Se_*x*_ samples with the increase of Se content. For SnS_2_ NSs^32^, only A_1g(S−Sn)_ mode was detected at 313.4 cm^−1^. The absence of intra-layer E_g(S−Sn)_ can be ascribed to the weak rejection of Rayleigh scattered radiation or the choice principle for scattering geometry in SnS_2_ nanosheets^18^,^34^. In contrast, two prominent vibration peaks in SnSe_2_ plates assigned to A_1g(Se−Sn)_ mode at 177.4 cm^−1^ and E_g(Se−Sn)_ mode at 99.2 cm^−1^ are observed. Because of the low concentration of Se in SnS_1.66_Se_0.34_ alloy, the vibration peaks of SnSe_2_-like modes are not strong enough to be observed. Similarly, the peaks of SnS_2_-like modes are also not obvious in SnS_0.44_Se_1.56_ with low S content. As shown in Fig. 3, the intensity of SnS_2_-like A_1g_ mode would decrease until completely disappear, while the SnSe_2_-like A_1g_ and E_g_ modes come into appearance and gradually enhance with increasing Se content. The peak patterns of pure material (SnS_2_^32^ or SnSe_2_) are simple and sharp, while that of the alloys are broad and complex. In addition, all the vibration modes shift to low frequency. The transitions of molecular vibration modes exhibit strong dependence on increasing Se concentrations in the composition-dependent SnS_2−*x*_Se_*x*_ alloys.

X-ray photoelectron spectroscopy (XPS) was used to measure the changes of binding energy of Sn, S and Se in SnS_2−*x*_Se_*x*_ alloys. As shown in Supplementary Fig. S6a, the binding energies of Sn 3d_5/2_ at 486.4 eV and Sn 3d_3/2_ at 494.8 eV in SnSe_2_ plates were close to that of pure SnS_2_ NSs^32^ (Sn 3d_5/2_ at 486.2 eV and Sn 3d_3/2_ at 494.6 eV). Compared with SnSe_2_, the peaks of Sn 3d_5/2_ and Sn 3d_3/2_ in SnS_0.44_Se_1.56_ present a shift of about 0.5 eV to 486.9 and 495.3 eV. As shown in Supplementary Fig. S6b, Se 3d_5/2_ and Se 3d_3/2_ peaks locate at the same peak positions of 54.2 and 54.9 eV for SnS_0.44_Se_1.56_ and SnSe_2_. The binding energies of S 2p_3/2_ (160.5 eV) and S 2p_1/2_ (161.6 eV) in SnS_0.44_Se_1.56_ (Supplementary Fig. S6c) were lower than that of pure SnS_2_ with S 2p_3/2_ at 161.1 eV and S 2p_1/2_ at 162.5 eV^32^. Meanwhile, the peaks from Se 3p_3/2_ at 158.9 eV and Se 3p_1/2_ at 166.4 eV become dominant, which is in agreement with previous work^14^. The obvious shift of binding energies in SnS_2−*x*_Se_*x*_ alloy indicated the formation of solid solutions, which was attributed to the easy electron transfer in SnS_2−*x*_Se_*x*_ alloy because of strong combination between different atoms^35^.

**Figure 4:**
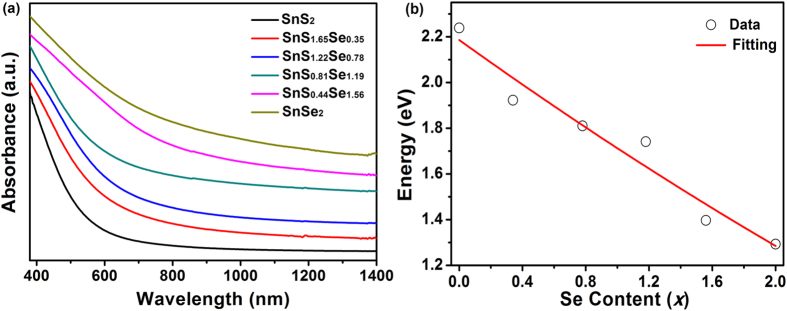
(**a**) UV-vis-NIR absorption spectra and (**b**) composition-dependent band gaps and the corresponding fitting curve of SnS_2−*x*_Se_*x*_ alloys.

The band gaps could be tuned by controlling the chemical compositions of SnS_2−*x*_Se_*x*_ alloys. Figure 4a shows the UV-vis-NIR absorption spectra of SnS_2−*x*_Se_*x*_ with different Se contents. The absorption edge exhibits a red-shift with the increase of Se content, indicating enhanced optical absorption property. The band gap of semiconductor could be calculated by extrapolating straight line of the plot (*αhν*)^1/2^ vs. *hν* based on the equation: *αhν* = *A*(*hν–E*_*g*_)^n/2^, and the estimated data were shown in Table 1. SnS_2−*x*_Se_*x*_ alloys own the band gap ranging from 2.23 eV for SnS_2_ to 1.92, 1.81, 1.74, 1.39 and 1.29 eV with increasing Se content. The empirical relation between band gap and composition ratio has been predicted according to the extended Vegard’s Law. Figure 4b presents the composition-dependent band gaps of SnS_2−*x*_Se_*x*_ alloys. The solid line represents the fitted values for the band gap relation of ternary semiconductor alloys according to the generalized equation^9^,^36^,^37^:


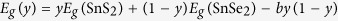


where *b* is the optical bowing constant. In this work, *y* equals to *x*/2. The best fit yields *b* = 0.03, which was found to be in the range of 0 to 0.65^30^. And the small *b* value demonstrates that SnS_2_ and SnSe_2_ have a good miscibility^24^,^38^. The non-linear relationship is ascribed to the alteration of band structure caused by the volume deformation in SnS_2−*x*_Se_x_ alloys and the change of electron distribution due to the electronegativity difference of different atoms^35^,^39^.

## Discussion

**Figure 5:**
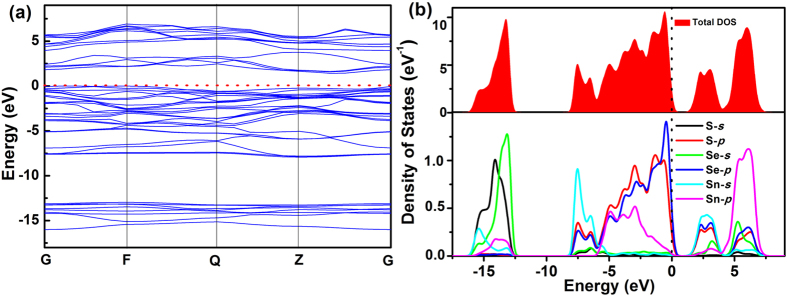
(**a**) Calculated band structure of SnS_0.44_Se_1.56_ alloy. (**b**) The total and partial density of states of SnS_0.44_Se_1.56_.

For further theoretical study, we employed first principles calculations to obtain the band gap structures and DOS curves, which are benefit to analyze the electronic structures of SnS_2−*x*_Se_x_ alloys and possible affecting factors. Figure 5 shows the first-principles calculations results of SnS_0.44_Se_1.56_ alloy. The corresponding band structure is shown in Fig. 5a, which clearly demonstrates SnS_0.44_Se_1.56_ is an indirect band gap semiconductor. The band gap of SnS_0.44_Se_1.56_ is calculated to be 1.421 eV, which is in good accordance with the experimental value of 1.39 eV. Additionally, the band gaps of pure SnS_2_ and SnSe_2_ were estimated to be 2.461 and 1.402 eV, respectively (Supplementary Fig. S7), which are close to the experimental values of 2.23 and 1.29 eV. The calculated results roughly reveal the variation tendency of band gaps with the increase of Se concentration in SnS_2−*x*_Se_*x*_ crystals, which might be ascribed to the replacement of S and Se, affecting electronic structure distribution in the alloy system^37^. The total and partial density of states (TDOS and PDOS) of SnS_0.44_Se_1.56_ are provided in Fig. 5b, and the energy zero is defined as Fermi energy level. From Fig. 5b, we could conclude the contribution of different orbitals to VB and CB SnS_0.44_Se_1.56_ alloy. PDOS curves actually presented different tendencies in the regions close to the VB and CB. The states near VB are dominated by the S 3*p* and Se 4*p* orbitals, while CB is mainly composed of hybridized states of Sn 5*s*, S 3*p* and Se 4*p* orbitals. The difference of constituting orbitals in VB and CB would result in the dissimilarities of band structure in SnS_2−*x*_Se_*x*_ alloys with the change of S/Se ratio.

In order to explore the potential applications of SnS_2−*x*_Se_*x*_ alloys in optoelectronic devices, the photoelectrochemical performances were carried out to study the separation and transmission efficiency of photogenerated electrons and holes. The photocurrent densities are 2.1 μA cm^−2^ for SnS_2_, 1.6 μA cm^−2^ for SnS_1.66_Se_0.34_, 3.3 μA cm^−2^ for SnS_1.22_Se_0.78_, 4.3 μA cm^−2^ for SnS_0.82_Se_1.18_, 27.2 μA cm^−2^ for SnS_0.44_Se_1.56_ and 14.1 μA cm^−2^ for SnSe_2_, respectively (see Table 1). As shown in Supplementary Fig. S8a, the photocurrent values presented negligible variation under continued irradiation of monochromatic green light (*λ* = 550 nm), clearly revealing the good photostability of SnS_2−*x*_Se_*x*_ alloys. After the incorporation of Se element, SnS_2−*x*_Se_*x*_ alloys presented gradually enhanced absorption regime with increasing Se doping (Fig. 4). Correspondingly, the photocurrent values of ternary materials presented significant and regular increase. The current density of SnS_0.44_Se_1.56_ still remained at 27.2 μA cm^−2^ after 500 s of irradiation. As is well known, the morphology, size, and spatial arrangement of materials have an important effect on their properties. From previous reports^2^,^40^, 2D configuration would endow semiconductor material a more convenient transmission approach for photogenerated electrons and holes and a much better grain boundary connectivity, which benefits to enhance electron−hole pairs transport/separation efficiency and prevents their recombination. Compared with SnS_0.82_Se_1.18_, the photocurrent of SnS_0.44_Se_1.56_ NSs presented tremendous enhancement from 4.31 μA cm^−2^ to 27.2 μA cm^−2^. Furthermore, the value is much larger than that of SnSe_2_ plates (14.1 μA cm^−2^), which owns narrower band gap than SnS_0.44_Se_1.56_ NSs. The stacked structures (SnS_0.82_Se_1.18_ and SnSe_2_) would provide a long approach to the surface for photogenerated electrons and holes. And before collected, inner carriers were easier to be lost because of recombination, which needed to take longer time to arrive at the surface than those generated near the surface^41^,^42^. Noteworthily, the current value of SnS_2_ (2.1 μA cm^−2^) is higher than that of SnS_1.66_Se_0.34_ (1.6 μA cm^−2^), which might be ascribed to the dense stacking of small nanosheets on ITO during the preparation of photoelectrode. The repeated ON/OFF switching measurements were carried out to examine the sensitivity of materials to incident light. As shown in Supplementary Fig. S9, the photoresponse currents of sheet-based materials would reach steady state with a short time, however, the stacked structures needed a longer response time. This delay phenomenon is particularly apparent for SnS_0.82_Se_1.18_. That could be attributed to the large contact area and thin thickness of 2D materials, which enable them to capture visible light efficiently and encourage electron-hole pairs to transfer fast. Accordingly, the recombination probability of photogenerated electrons and holes would be reduced to a low level. The corresponding electrochemical impedance spectra (Supplementary Fig. S8b) present analogous variation tendency to the *I*–*t* curves. Lower EIS means smaller interfacial charge–transfer resistance. SnS_0.44_Se_1.56_ NSs exhibit the lowest EIS, which greatly benefits to carrier transport efficiency in the electrode.

**Figure 6:**
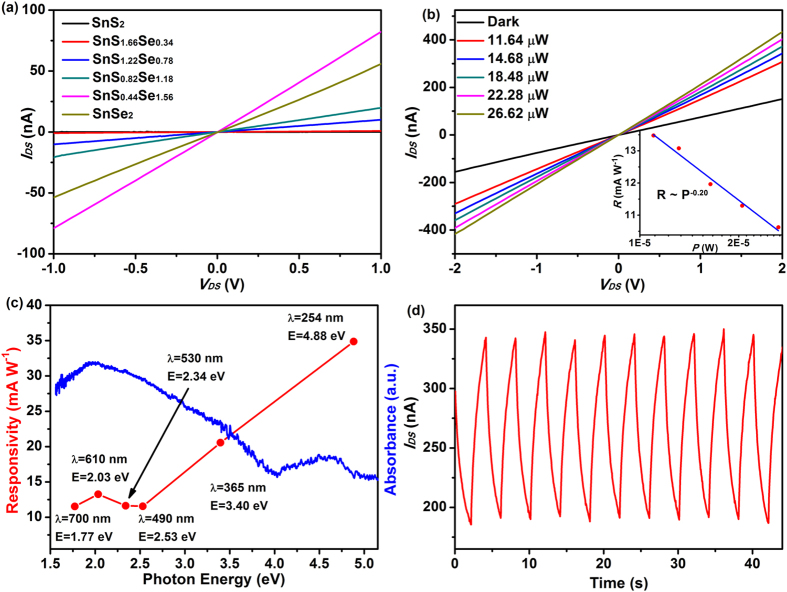
Electrical and photodetector properties of SnS_2−*x*_Se_*x*_ alloys. (**a**) *I*_*DS*_–*V*_*DS*_ curves for the devices. The linearity indicates excellent Ohmic contacts in the SnS_2−*x*_Se_*x*_ devices. (**b**) *I*_*DS*_–*V*_*DS*_ curves for SnS_0.44_Se_1.56_ device with various illumination power *P* (*λ* = 610 nm). The inset shows the logarithmic scale plot of photoresponsivity *R* as a function of light power (**c**) The photoresponsivity *R* of SnS_0.44_Se_1.56_ device at different illumination wavelengths (*P* = 16.36 μW) with a bias voltage of 2 V (red line) and solid phase UV-vis absorption spectrum of SnS_0.44_Se_1.56_ alloy (blue line). (**d**) The time trace of photocurrent response for SnS_0.44_Se_1.56_ device at a bias voltage of 2 V (*λ* = 610 nm, *P* = 16.36 μW).

Tin-based chalcogenides have been widely studied as the building blocks for nanoelectronics^21^,^43^,^44^,^45^, which would provide great potentials for next-generation electronic applications. For further extending the optoelectronic applications, we measured the optoelectronic response of as-prepared SnS_2−*x*_Se_*x*_ in a wide range from ultraviolet to visible light. A schematic depiction of the devices structure is shown in Supplementary Fig. S10, and the results of electrical and photodetector properties are presented in Fig. 6 and Table 1. At low source-drain voltage, the *I*_*DS*_–*V*_*DS*_ curves of all devices are symmetric and linear, indicating the Ohmic contacts between Au electrodes and SnS_2−*x*_Se_*x*_ films. The currents present significant enhancement with the increasing Se content, which might be attributed to the synergistic influence of morphology and tunable electronic structure. The SnS_0.44_Se_1.56_ film shows highest current values. Its unique 2D configuration (large dimension of 1.8−2.5 μm and thin thickness of ca. 8 nm) would provide more active sites and shorter route in electronic transfer process. Furthermore, the photoresponse measurements for SnS_0.44_Se_1.56_ alloy were carried out. Figure 6b provides *I*_*DS*_ vs *V*_*DS*_ curves of SnS_0.44_Se_1.56_ device without and with red light illumination (*λ* = 610 nm) with various power intensity. With increasing power intensity, the photocurrent distinctly increases, which could be ascribed to the increasing number of photogenerated carriers. The photoresponsivity *R*, defined as the ratio between photocurrent increase (Δ*I*) and power intensity (*P*), Δ*I*/*P,* as a function of illumination power is shown in the inset of Fig. 6b. It is clear that *R* decreases with the increase of laser power, which may be attributed to the enhanced scattering or recombination rate of hot carriers at higher laser power intensity^20^. The relationship between photoresponsivity versus incident light power can be fitted by a power law relationship *R* ∝ *P*^α−1^ 20,46,47. The fitting parameter α = 0.80 was obtained in our measurement, which is comparable to that of layered SnS_2_ (α = 0.77)^20^ and few-layer MoS_2_ (α = 0.71)^46^, indicating that the recombination kinetics of photogenerated carriers is related to both trap states and interaction of carriers^47^.

The spectral responsivity of SnS_0.44_Se_1.56_ device as a function of illumination wavelength is presented in Fig. 6c and Supplementary Fig. S11. Large photocurrents could be observed at high light energy (34.9 mA W^−1^ for photon energy *E*_*ph*_ = 4.88 eV and 20.6 mA W^−1^ for *E*_*ph*_ = 3.40 eV). Interestingly, the photoresponsivity was not continuous enhanced with the increase of excitation energy. In the visible region, the largest photocurrent of 13.3 mA W^−1^ was obtained at *E*_*ph*_ = 2.03 eV (*λ* = 610 nm), in accordance with the absorption spectrum of SnS_0.44_Se_1.56_, where it possesses the strongest photoabsorption abilities at *λ* = 632 nm. The results suggest that the photocurrent is derived from the absorption of light energy and the fast generation and separation process of electron-hole pairs in SnS_0.44_Se_1.56_ layers. It deserves to be noted that the photoresponsivity in our work is higher than the reported values in previous literatures (8.8 mA W^−1^ for layered SnS_2_^20^, 92 μA W^−1^ for multilayer WS_2_ films^48^, 7.5 mA W^−1^ for MoS_2_^49^). Furthermore, we calculate the external quantum efficiency (EQE) of SnS_0.44_Se_1.56_ photodetector, which represents the number ratio of photogenerated carriers to incident photons. EQE could be estimated according to the equation: EQE = *hcR*/(*eλ*), in which *h* means the Planck’s constant, *c* is the speed of light, *λ* is the incident light wavelength, and *e* means the electron charge. The EQE of our device was estimated to be 2.69% at *P* = 16.36 μW and *λ* = 610 nm, which is a little higher than that of layered SnS_2_ obtained by CVD growth (EQE = 2.4%)^20^. In addition, time-resolved photoresponse behavior of the device was carried out and shown in Fig. 6d. The device exhibits an obvious current change (~180 nA) and good stability after a long-time operation with alternated turn-on and turn-off process. The long response time might be ascribed the combination of extrinsic traps, such as adsorbates at the SnS_0.44_Se_1.56_ surface and SiO_2_/Si substrate, or the contact barrier of SnS_0.44_Se_1.56_ film and, as well as intrinsic factors, including defect states in the SnS_0.44_Se_1.56_ itself prepared by solution synthesis process^17^.

In summary, ternary SnS_2−*x*_Se_*x*_ alloys with tuneable composition (0 ≤ *x* ≤ 2) have been prepared *via* a simple one-step solvothermal procedure. The crystal structures and morphologies of alloys presented large difference with the change of doped Se content. The lattice constant *a* and optical properties are found to be composition-dependent and could be tuned by altering S/Se ratio. Their band gaps would change from 2.23 to 1.29 eV, which is in line with the extended Vegard’s Law. The first-principles calculations theoretically proved the tunability of band structure in SnS_2−*x*_Se_*x*_ alloys, and the calculation results are in consistent with experimental values. PDOS curves indicate that the VB and CB of ternary alloys are derived from different atom orbitals. SnS_0.44_Se_1.56_ NSs exhibit outstanding photoresponse behavior under the irradiation of green light. The PEC performances of materials not only just depend on their band structures, but also their morphologies. Furthermore, the high photoresponsivity (a maximum of 35 mA W^−1^) in a wide range of spectra, combined with their optical stability in SnS_2−*x*_Se_*x*_ devices can be attractive for a variety of optoelectronic applications.

## Methods

### Synthesis of SnS_2−*x*_Se_*x*_ Alloys

All chemicals were of analytical grade and used as received without further purification. Tin (II) chloride dihydrate (SnCl_2_•2H_2_O), thioacetamide (TAA, C_2_H_5_NS), selenium dioxide (SeO_2_) and triethylene glycol (TEG, C_6_H_14_O_4_) were obtained from Sinopharm Chemical Reagent Co., Ltd. Polyvinylpyrrolidone (PVP, *M*_*w*_ = 55000) was purchased from Sigma-Aldrich.

SnS_2−*x*_Se_*x*_ nanosheets and nanosheets assemblies were prepared by solvothermal synthesis, similar to that for SnS_2_ nanosheets (NSs)^32^. SeO_2_ was used as Se source. For the synthesis of SnS_2_ NSs, 1 mmol (0.2257 g) SnCl_2_•2H_2_O, 2 mmol (0.1503 g) TAA and 0.5 g PVP were added into 30 mL of TEG. After complete dissolution through vigorous magnetic stirring at room temperature, the precursor solution was transferred into a 50 mL Teflon-lined stainless steel autoclave. The autoclave was heated at 220 °C for 12 h and then cooled down to room temperature naturally. The precipitate was centrifuged at 10, 000 rpm for 8 minutes and washed several times with deionized water and absolute ethanol, respectively. The final product was collected after dried at 60 °C overnight. SnSe_2_ plates were obtained by adding 2 mmol (0.2219 g) SeO_2_ as Se source instead of TAA. However, there would be little Se residue because of the low solubility of SeO_2_.

Ternary alloys were prepared by modulating the ratio of S and Se atoms with 2 mmol mixture of TAA and SeO_2_ powders. The initial S/Se molar ratio was set as 8:2, 6:4, 4:6 and 2:8. The obtained samples were denoted as SnS_2−*x*_Se_*x*_, where *x* represented the molar ratio of doping Se.

### Characterization

The crystal structure of samples was determined by Rigaku D/max-IIIB X-ray diffraction (XRD) (Cu K_α_ irradiation, *λ* = 1.54178 Å). Scanning electron microscope (SEM) and energy-dispersive spectroscopy (EDS) measurement were used to examine the morphology and chemical composition of as-obtained products on FEI Quanta 200F microscope. The thicknesses of SnS_2−*x*_Se_*x*_ nanosheets and nanosheets assemblies were measured by Bruker Dimension ICON-Pt atomic force microscopy (AFM). TEM and selected area electron diffraction (SAED) were also recorded on FEI Tecnai G^2^ F30 TEM. The absorption spectra were recored on PerkinElmer Lambda 950 UV/vis/NIR spectrometer. Raman spectra were measured on a LaBRAM HR800 (Jobin Yvon Horiba) Raman spectrometer with a He-Ne laser (*λ* = 532 nm). X-ray photoelectron spectroscopy (XPS) measurement was characterized on Thermo Fisher Scientific VG K_α_ Probe spectrometer.

### Photoelectrochemical Measurements

Photoresponse behaviour of as-synthesized samples was carried out on a conventional three-electrode configuration with CHI 660E electrochemical workstation. A Pt wire and Ag/AgCl electrode were used as the counter and reference electrodes, respectively. 1 × 1 cm^2^ ITO conductive glass coated by dropping 1 mL 1.5 mg mL^−1^ of sample was used as work electrode. 0.5 M Na_2_SO_4_ aqueous solution (pH = 6) was used as electrolyte in all electrochemical tests. A 300W Xe lamp (CEL-HXF 300, Beijing Au-light, China, *I* = 20 A) with the monochromatic green light (*λ* = 550 nm) was employed as light source with distance of 10 cm to the photoelectrode placed in quartz cell. The current-time (*I-t*) curves were recorded with a bias potential of 0.5 V *vs*. Ag/AgCl electrode. Electrochemical impedance spectroscopy (EIS) was measured with a frequency of 100 kHz–100 mHz at a bias potential of 0.5 V *vs.* Ag/AgCl electrode.

### Fabrication and Measurements of Photodetector Devices

The SnS_2−*x*_Se_*x*_ films were prepared onto the Si substrate with a 300-nm-thick SiO_2_ dielectric layer. Then, the Au electrodes with thickness of 120 nm were deposited by electron-beam evaporation with help of shadow mask, and the channel length was about 2 mm. The electrical measurements were performed under ambient conditions using a Keithley semiconductor parameter analyzer, model 4200-SCS. The monochromatic light with different wavelengths was applied, and the power intensity was calibrated by a power meter.

## Additional Information

**How to cite this article**: Yu, J. *et al.* Ternary SnS_2–*x*_Se_*x*_ Alloys Nanosheets and Nanosheet Assemblies with Tunable Chemical Compositions and Band Gaps for Photodetector Applications. *Sci. Rep.* **5**, 17109; doi: 10.1038/srep17109 (2015).

## Supplementary Material

Supplementary Information

## Figures and Tables

**Table 1:** Compositions, lattice parameter *a*, band gaps and photodetector properties of SnS_2−*x*_Se_*x*_ alloys.

Theoretical *x*	0	0.4	0.8	1.2	1.6	2
Experimental *x*	–	0.34	0.78	1.18	1.56	–
(S + Se)/Sn	1.88	2.18	2.20	2.14	2.07	1.96
Lattice parameter *a* (±0.01 Å)	3.649	3.663	3.709	3.756	3.790	3.812
*E*_*g*_ (eV)	2.23	1.92	1.81	1.74	1.39	1.29
Photocurrent (μA cm^−2^)	2.1	1.6	3.3	4.3	27.2	14.1
Current (nA)	0.05	0.9	10.0	19.8	82.3	55.9
