# Supplementary material for: Ternary SnS2–xSex Alloys Nanosheets and Nanosheet Assemblies with Tunable Chemical Compositions and Band Gaps for Photodetector Applications
Source: Sci Rep. 2015 Nov 30;5:17109. doi: 10.1038/srep17109 (PMC4663750; doi:10.1038/srep17109)
Supplement: Supplementary Information [file srep17109-s1.pdf]

Supplementary Information

Ternary $SnS_{2-x}Se_x$ Alloys Nanosheets and Nanosheet Assembly with Tunable Chemical Compositions and Band Gaps for Photodetector Applications

Jing Yu[1,2], Cheng-Yan Xu[1,2], Yang Li[1,2], Fei Zhou[1], Xiao-Shuang Chen[2,3], Ping-An Hu[1,2] & Liang Zhen[1,2]

[1]School of Materials Science and Engineering, Harbin Institute of Technology, Harbin 150001, China, [2]MOE Key Laboratory of Micro-systems and Micro-structures Manufacturing, Harbin Institute of Technology, Harbin 150080, China, [3]Department of Physics, Harbin Institute of Technology, Harbin 150001, China

Correspondence and requests for materials should be addressed to C.X. (cy_xu@hit.edu.cn) and L.Z. (lzhen@hit.edu.cn)

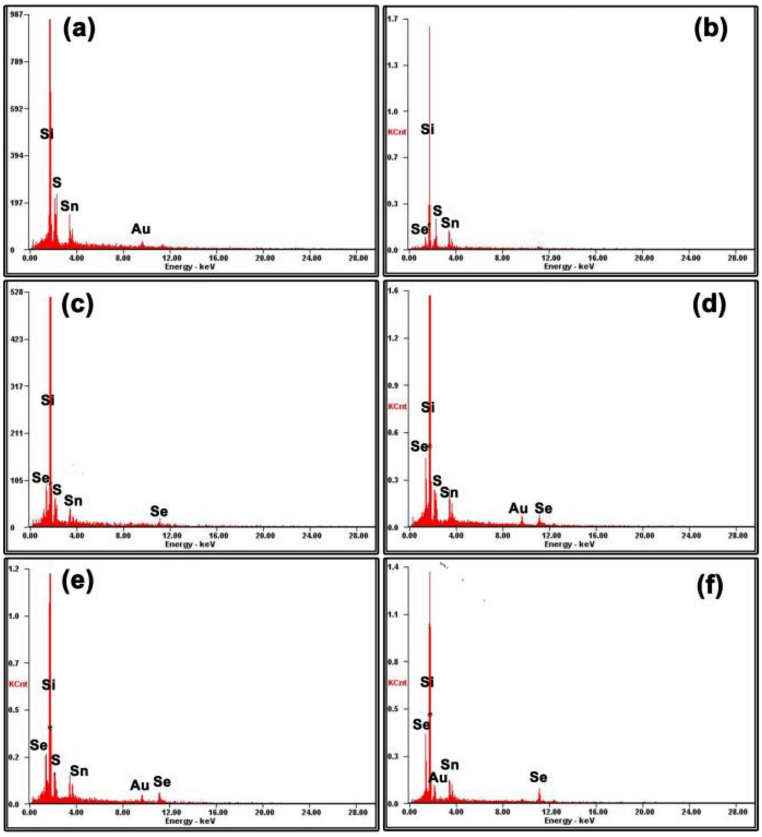

Fig. S1. EDS of as-prepared $SnS_{2-x}Se_x$ alloys with different x values. (a) SnS_2. (b) $SnS_{1.66}Se_{0.34}$. (c) $SnS_{1.22}Se_{0.78}$. (d) $SnS_{0.82}Se_{1.18}$. (e) $SnS_{0.44}Se_{1.56}$. (f) $SnSe_2$.

Table S1 Crystallite dimension of all the samples derived from Scherrer equation.

Sample	SnS_2	$SnS_{1.66}Se_{0.34}$	$SnS_{1.22}Se_{0.78}$	$SnS_{0.82}Se_{1.18}$	$SnS_{0.44}Se_{1.56}$	$SnSe_2$
FWHM	0.648	0.796	0.688	0.662	0.993	0.335
D (nm)	12.4	9.9	11.5	12.0	7.9	23.6

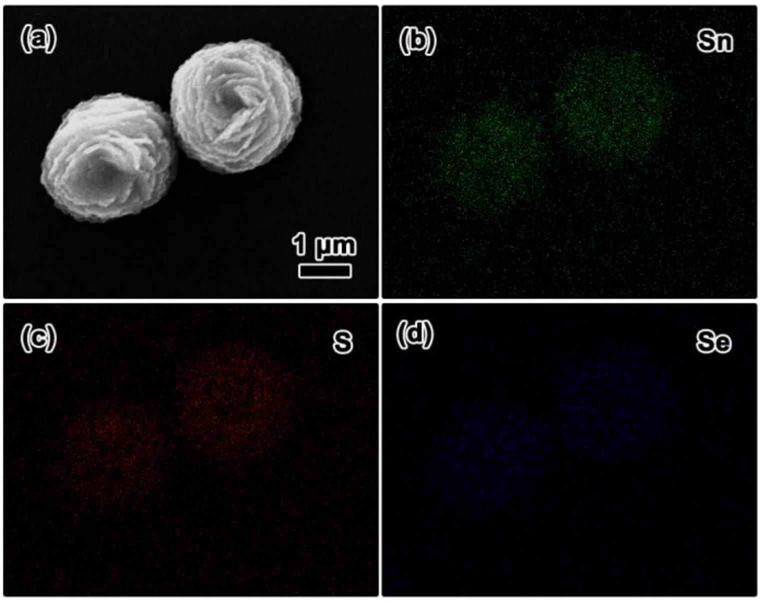

Fig. S2. EDS elemental mapping of $SnS_{0.82}Se_{1.18}$ alloy.

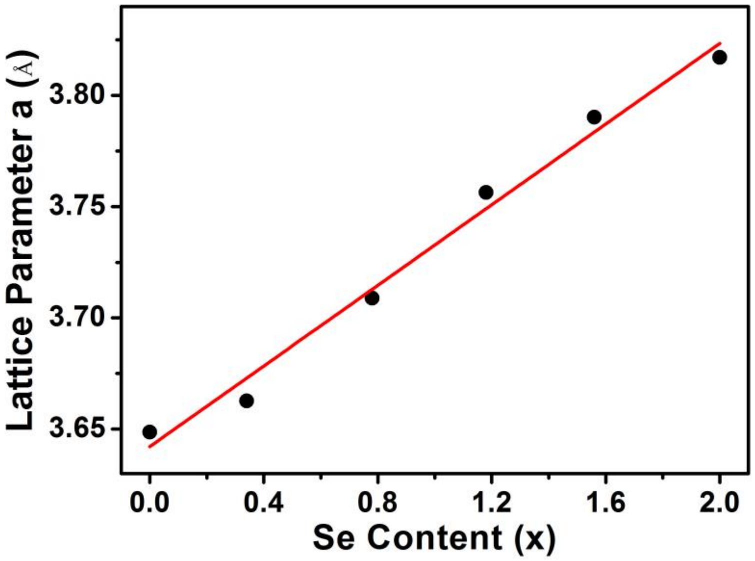

Fig. S3. Linear relationship of the lattice parameter *a* of $SnS_{2-x}Se_x$ as a function of *x* value.

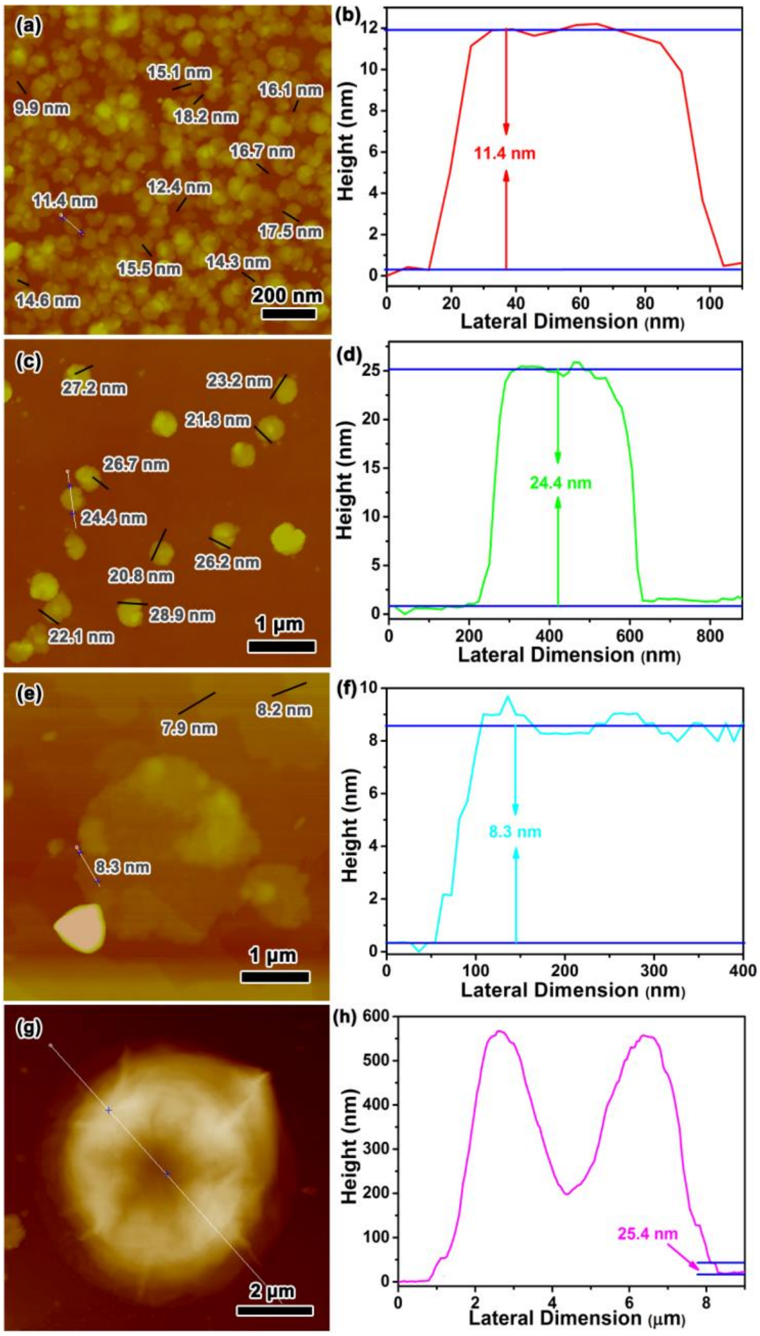

Fig. S4. AFM images and corresponding height profiles from individual nanosheets of $SnS_{2-x}Se_x$ alloys. (a, b) $SnS_{1.66}Se_{0.34}$. (c, d) $SnS_{1.22}Se_{0.78}$. (e, f) $SnS_{0.44}Se_{1.56}$. (g, h) $SnSe_2$.

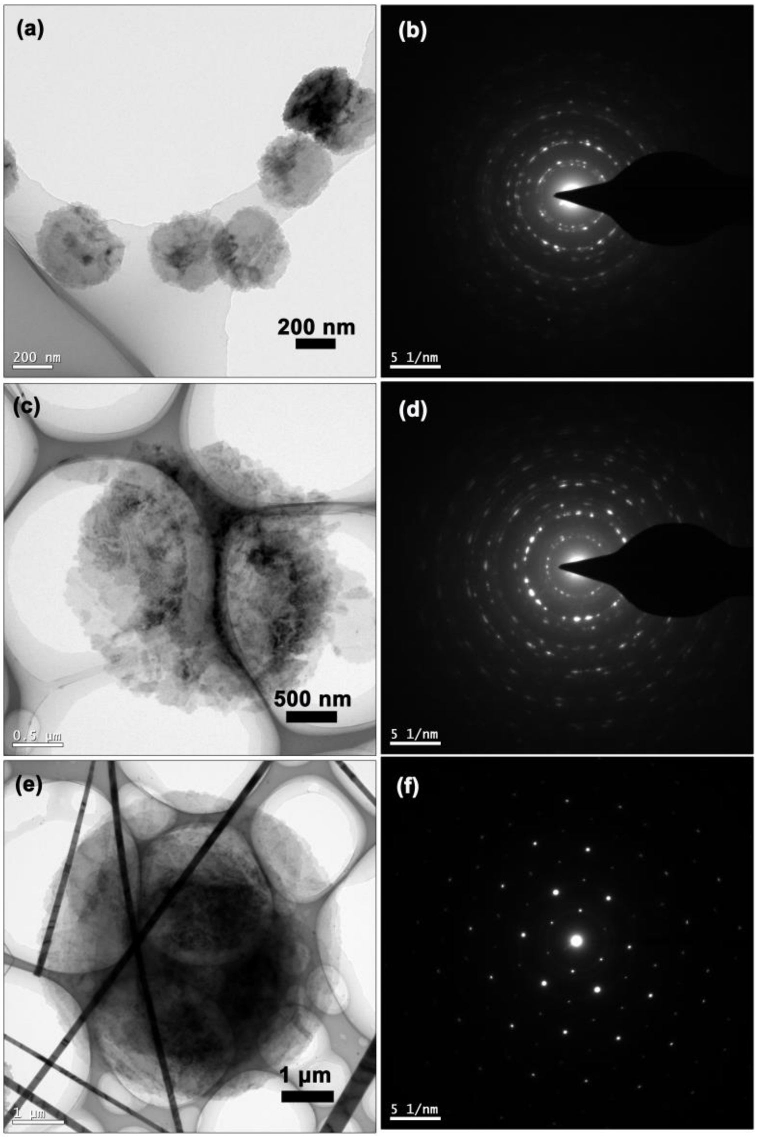

Fig. S5. TEM images and SAED patterns of as-prepared samples. (a, b) $SnS_{1.22}Se_{0.78}$. (c, d) $SnS_{0.44}Se_{1.56}$. (e, f) $SnSe_2$. The wires in (e) were considered as residual Se.

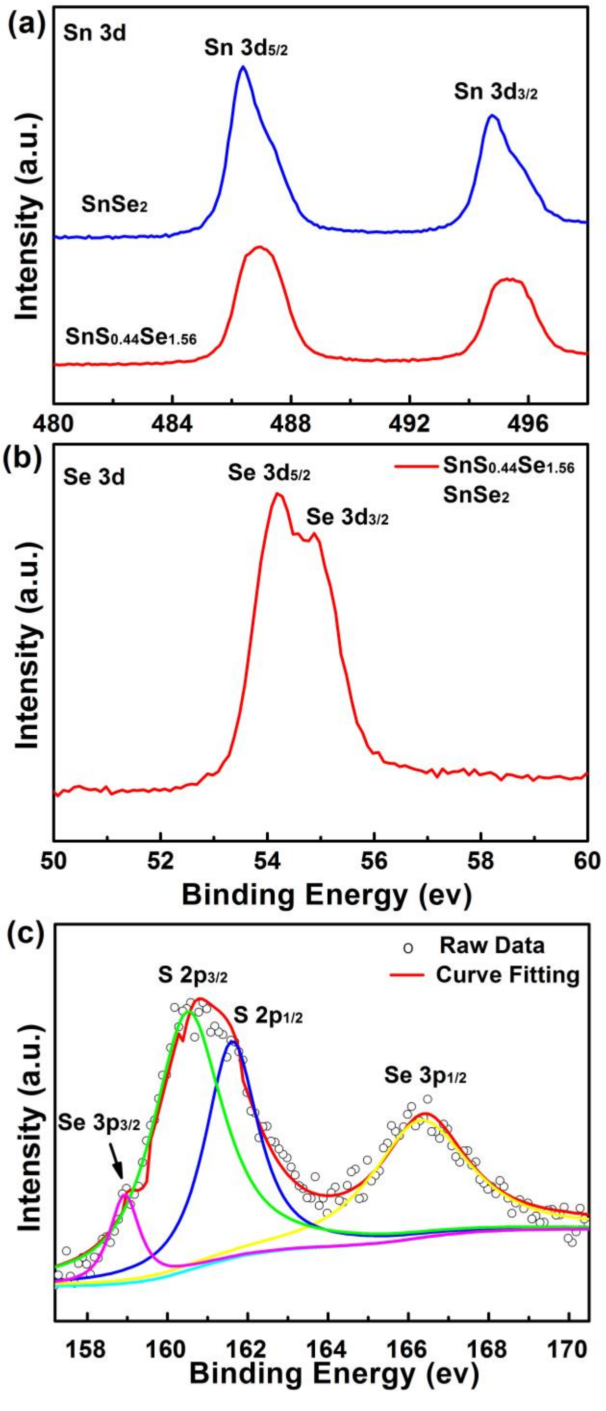

Fig. S6. XPS spectra of Sn 3d (a) and Se 3d regions (b) in $SnS_{0.44}Se_{1.56}$ and $SnSe_2$, respectively. (c) XPS of S 2p region in $SnS_{0.44}Se_{1.56}$ alloy.

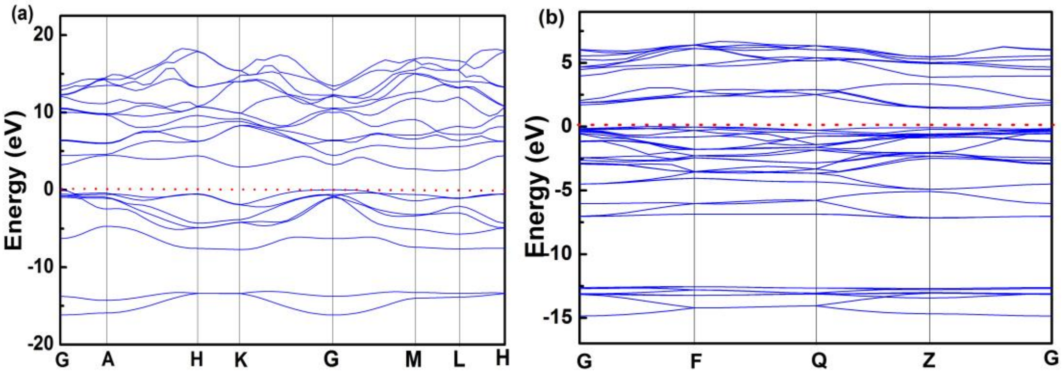

Fig. S7. Calculated band structures of pure SnS_2 (a) and $SnSe_2$ (b).

SnS_2 and $SnSe_2$ are both n-type semiconductors and possess indirect band gaps, which were calculated to be 2.461 and 1.402 eV, respectively. The results are in agreement with the experimental band gap values of 2.23 eV for $SnS_2$and 1.29 eV for $SnSe_2$.

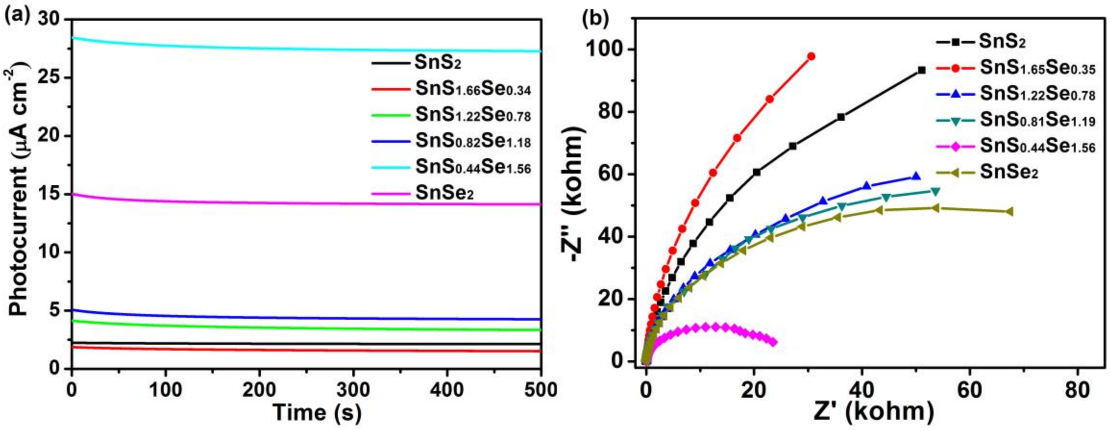

Fig. S8. (a) *I*-*t* curves of $SnS_{2-x}Se_x$ alloys at 0.5 V *versus* Ag/AgCl electrode under 300 W Xe lamp illumination (λ = 550 nm). (b) Electrochemical impedance spectra of the samples.

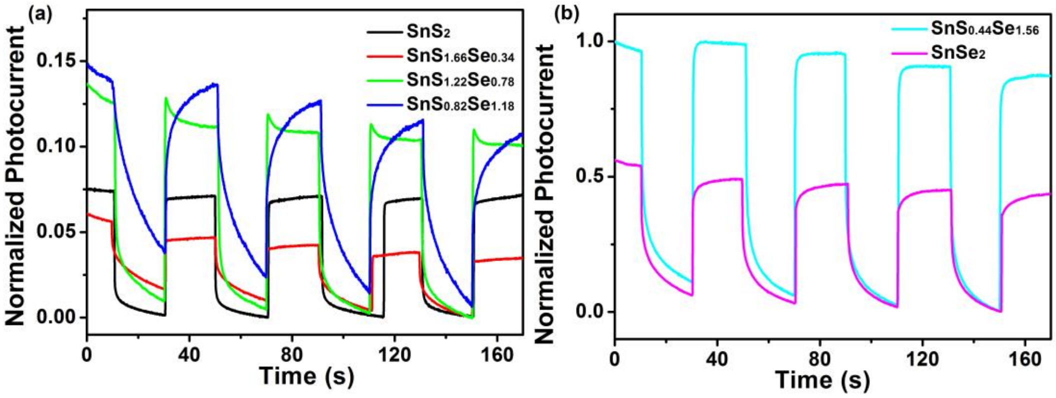

Fig. S9. *I*-*t* curves of $SnS_{2\text{-}x}Se_x$ alloys at 0.5 V *versus* Ag/AgCl electrode under 300 W Xe lamp illumination (λ = 550 nm).

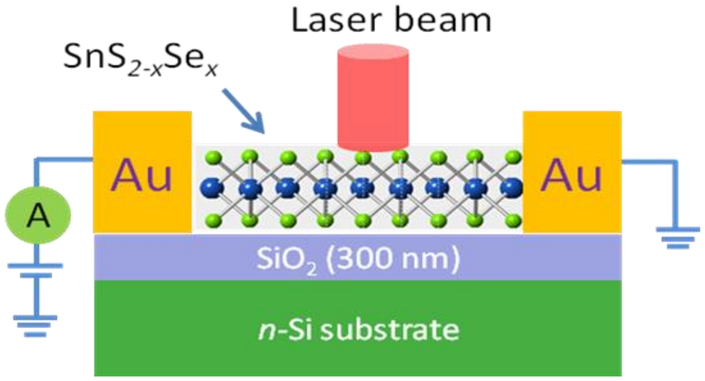

Fig. S10. Schematic diagram of the $SnS_{2-x}Se_x$ devices configuration.

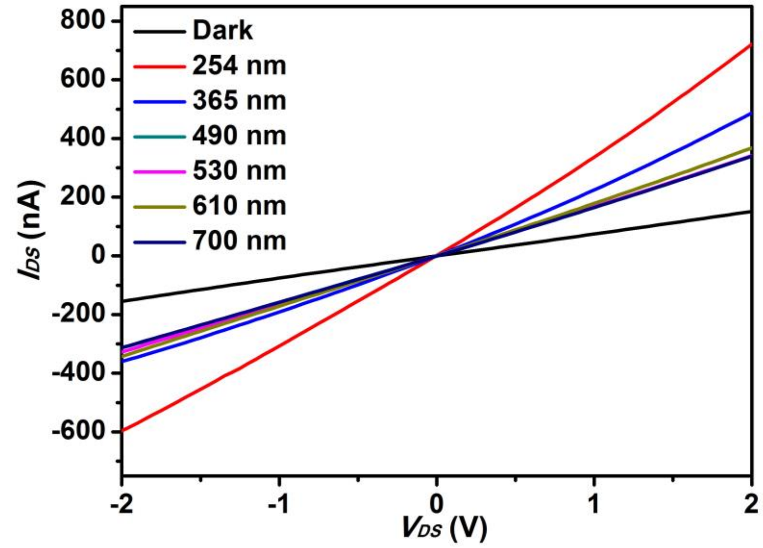

Fig. S11. I_{DS}–V_{DS} curves for $SnS_{0.44}Se_{1.56}$ device with various illumination wavelengths (P = 16.36 μW).

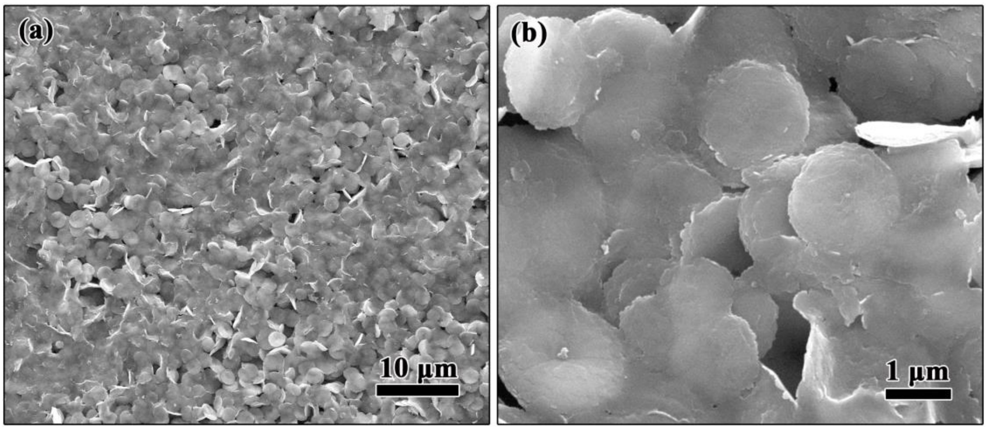

Fig. S12. SEM images of $SnS_{0.44}Se_{1.56}$ photophotodetector surface. The special 2D configuration and uniform distribution would provide a convenient and short route for the charge transfer.
